# Prevalence and Severity of Ectopic Eruption of First Permanent Molars in Pediatric Patients in Makkah, Saudi Arabia

**DOI:** 10.7759/cureus.49220

**Published:** 2023-11-22

**Authors:** Rahaf M Alotaibi, Sufana H Alattas, Banan N Alandanusi, Shahd S Alomiri, Tariq S Nazer, Ali M Alzahrani, Hanadi S Lingawi

**Affiliations:** 1 Dentistry, Umm Al-Qura University, Makkah, SAU; 2 Pediatric Dentistry, Security Forces Hospital, Makkah, SAU; 3 Pediatric Dentistry, Umm Al-Qura University, Makkah, SAU

**Keywords:** prevalence, dental anomalies, root resorption, first permanent molar, ectopic eruption

## Abstract

Background

Early detection and management of ectopic eruption (EE) of first permanent molars (FPMs) are crucial to avoid complicated treatments later.

Aim

This study aimed to assess the prevalence and severity of EE of FPMs among children in Makkah, Saudi Arabia.

Methods

This retrospective study was based on a radiographic evaluation of 1,008 dental panoramic radiographs performed for children attending the Dental Educational Hospital at Umm Al-Qura University and the Security Forces Hospital in Makkah. Patients' age, sex, tooth location, and severity of EE were assessed. The study adhered to the Strengthening the Reporting of Observational Studies in Epidemiology (STROBE) guideline for cross-sectional studies.

Results

Of the 1,008 reviewed cases, 18 (1.79%) were diagnosed with EE of FPMs. Among the 11 male patients, 81.82% showed severe EE, while 57.14% exhibited moderately severe EE among the seven female patients. The prevalences of EE in the maxilla and mandible were 1.59% and 0.20%, respectively. In contrast, the occurrence of EE of FPMs was similar between the right and left sides.

Conclusion

In this study, the prevalence of EE of FPMs among children in Makkah was 1.79%. The frequency and severity were both greater in male patients compared to female patients. While significantly more EE of FPMs was observed in the maxilla than in the mandible, there was no significant difference between the right and left sides.

## Introduction

The dental eruption is a tooth's journey to reach its final position in the mouth. It is a complex process and can often be interrupted or disturbed by many factors, including genetic, cellular, molecular, or tissue factors. The term ectopic eruption (EE) is usually used to describe the eruption of a tooth into an atypical position. It is essential to distinguish between EE and impaction, which occurs when a tooth cannot erupt because something is obstructing it and not because of its atypical position. The teeth most affected by EE are the maxillary first permanent molars (FPMs) and canines, the mandibular canines, the mandibular second premolars, and the maxillary lateral incisors in sequence [1]. The prevalence of EE of FPMs varies from 0.75% to 6% worldwide, depending on the population [2]. Although the exact etiology of EE of FPMs is unclear, it is considered multifactorial and includes genetic and local factors. Studies have revealed that EE of permanent molars may be caused by a small dental arch, large teeth, a deficiency in bony growth (particularly at the maxillary tuberosity area), early/premature tooth loss, or retained deciduous teeth [3, 4]. It is also often found in association with other dental anomalies, such as infra-occlusion of primary molars, small maxillary lateral incisors (peg laterals), congenitally missing second premolars, and EE of permanent canines [5]. In addition, the increased prevalence of EE of FPMs among siblings indicates a genetic link [6]. To prevent the consequent development of malocclusions, early detection of EE of FPMs is essential and can be achieved via a thorough examination of routine radiographic images taken for children between the ages of five and seven years (i.e., before the eruption of the FPMs). Dentists should also anticipate EE of FPMs when there is a delay of more than six months or when at least one FPM has an abnormal eruption position compared with the other FPMs [1, 6]. Various techniques have proven to be successful in treating EE of FPMs, for example, interproximal wedging and distal tipping of the FPM. However, selecting the right technique is critical. Usually, it depends on the patient's age, the second deciduous molar's condition, the second premolar's presence, and the severity of the case [7-9]. To the best of our knowledge, no studies have assessed the prevalence and characteristics of EE of FPMs in Makkah, Saudi Arabia. Thus, the aim of this study was to assess the prevalence and severity of EE of FPMs among children in Makkah, Saudi Arabia.

## Materials and methods

Study design and ethical considerations

This study utilized a retrospective cross-sectional design. It was based on the radiographic evaluation of dental panoramic radiographs (orthopantomogram (OPGs)) performed for pediatric patients aged 6-10 years old who attended the Dental Educational Hospital at Umm Al-Qura University (UQUDENT) and the Security Forces Hospital in Makkah, Saudi Arabia. The study protocol was approved by the Biomedical Research Ethics Committee of Umm Al-Qura University (approval number HAPO-02-K-012-2023-01-1405) and the Institutional Review Board of the Security Forces Hospital in Makkah (IRB number 0615010823). The study adhered to the Strengthening the Reporting of Observational Studies in Epidemiology (STROBE) guideline for cross-sectional studies. 

Study population

The study population consisted of healthy children aged 6-10 years old who attended the dental clinic at either UQUDENT or the Security Forces Hospital in Makkah between 2017 and 2022 and for whom a good, diagnostic-quality OPG had been taken. Children who met any of the following criteria were excluded from this study: history of any syndrome or craniofacial malformation; systemic condition with oral impacts; congenital anomaly; missing primary second molar(s); tooth loss due to trauma, caries, or orthodontic treatment; or poor-quality OPG. Data were collected on the age and sex of the participants, the tooth number, the arch, the location of ectopically erupted FPMs, and the severity of resorption of the roots of the primary molars.

Radiographical assessment

Data collection was performed by four general dentists who had previously undergone training and calibration in radiographic diagnosis, and the classification of the severity of EE of FPMs according to the criteria listed in Table 1 [2,10]. The four general dentists separately visually examined and interpreted the radiographic images to determine the presence and severity of any cases of EE of FPMs. Inter-examiner reliability was determined using Cohen's kappa coefficient prior to study commencement, and a high level of reliability (K = 0.87) was documented. A total of 1,008 participants fulfilled the selection criteria and were included in this study. The participants' OPGs were anonymized using serial numbers, and no names were included; thus, the participants could not be identified. The cases were divided equally between the four general dentists, and all the OPGs were examined using the same screen resolution and brightness. Information on each of the following parameters was recorded in the data collection form: serial number, gender, age, ectopic tooth number, site, and arch. The severity of the EE of any FPMs was recorded based on the effect on the second primary molars, as shown in Table 1. 

**Table 1:** Classification system used to record the severity of ectopic eruption. Source: [2, 10]

Criterion	Definition
Mild	Limited resorption to the cementum or with minimum dentine penetration.
Moderate	Resorption of the dentine without pulp exposure.
Severe	Resorption of the distal root or mesial root, leading to pulp exposure.

Statistical analysis

SPSS version 23.0 (IBM Corp., Armonk, NY, USA) software was used to assess the descriptive statistics of the parameters. Means and SDs were included in the statistical analysis. The χ2 test was used to assess the frequency distribution of the ectopically erupted FPMs according to age, gender, arch, site, and severity. Spearman's correlation was used to assess associations among age, gender, and EE severity. The confidence level was set to 95%, and p-values less than 0.05 were considered significant.

## Results

Four general dentists reviewed a total of 3,192 cases, and 1,008 (31.58%) cases met the inclusion criteria and thus formed the study sample. Of the included cases, 47.96% were male and 52.04% were female. The participants ranged in age from 6 to 10 years, and the mean age was 8.53 ± 1.62 years. In terms of gender, the mean age of the female patients was 8.42 ± 1.6 years, and the mean age of the male patients was 8.64 ± 1.64 years (Table 2).

**Table 2:** Mean age and SD of the study sample.

Category	Mean age (years)	SD (years)
Male	8.64	1.64
Female	8.42	1.6
Total	8.53	1.62

In our study sample, 18 patients were diagnosed with EE of FPMs, representing a prevalence of 1.79%. Among the patients diagnosed with EE of FPMs, 11 were male (2.28%), and seven were female (1.34%). A Chi-square test (χ2 ) was conducted to assess the relationship between the prevalence of EE and gender, and the resultant p-value was 0.374. In terms of the prevalence of EE in each jaw, a distinct pattern was revealed. The prevalence of EE in the maxillary arch was 1.59%, with 16 occurrences, while the prevalence of EE in the mandibular arch was notably lower at 0.20%, with only two occurrences (p <0.001) (Table 3).

**Table 3:** Prevalence of ectopic eruption of first permanent molars according to gender and jaw type.

Category	Ectopic eruption N (%)	P-value
Gender		0.374
Male	11 (1.1%)	
Female	7 (0.69%)	
Total	18 (1.79%)	
Arch		<0.001
Maxillary arch	16 (1.59%)	
Mandibular arch	2 (0.20%)	

As shown in Table 4, the severity of the observed EEs markedly differed between the male and female patients. In the male patients, the majority of cases (81.82%) were classified as severe, whereas in the female patients, the majority of cases (57.14%) were moderate and mild (14.29%). No cases of mild EE of FPMs were observed among the male patients. A χ2 test was conducted to evaluate the relationship between EE severity and gender, and the resultant p-value of 0.063 suggested that there was no statistically significant relationship between EE severity and gender.

**Table 4:** The relationship between the severity of ectopic eruption of first permanent molars and gender.

Severity level	Male (%)	Female (%)	P-value
Mild	0.00%	14.29%	0.063
Moderate	18.18%	57.14%	
Severe	81.82%	28.57%	

In contrast, the occurrence of EE of FPMs was similar between the right and left sides. A χ2 test was conducted to determine whether there was a significant association between the side of the mouth (where EE was present) and gender. The resultant p-value of 0.405 indicated that the distribution appeared to be similar in both the male and female patients (Table 5).

**Table 5:** Contingency table for side of ectopic eruption of first permanent molars and gender.

Side	Male	Female
Right and left	8	3
Left	2	2
Right	1	2

An ordinal logistic regression model was applied, and as shown in Table 6, the results revealed that there was no significant difference in the severity of EE of FPMs when the right and left sides were compared to both sides (p = 0.724 and 0.693, respectively). This indicated that the side of occurrence did not play a significant role in the severity of the observed EE.

**Table 6:** Ordinal logistic regression model for the relationship between side and arch and severity of ectopic eruption.

Description	Coefficient	Std. error	z-value	P-value	95% CI
Right side (vs. both sides)	-0.5211	1.475	-0.353	0.724	-3.412, 2.370
Left side (vs. both sides)	-0.4502	1.139	-0.395	0.693	-2.683, 1.783
Lower arch (vs. upper arch)	-0.3196	1.476	-0.217	0.829	-3.212, 2.572
Transition from mild to moderate	-3.0679	1.128	-2.72	0.007	-5.279, -0.857
Transition from moderate to severe	0.876	0.416	2.104	0.035	0.060, 1.692

Similarly, when the EE severity was examined in terms of the upper and lower arches, no significant difference in severity was found, as evidenced by the p-value of 0.829. 

## Discussion

Dental anomalies are not rare findings during routine dental examinations. However, their prevalence and severity vary among populations. Late detection of dental anomalies can complicate dental treatment and result in the need for multidisciplinary and more expensive treatment. The present study aimed to investigate the prevalence and severity of EE of FPMs among children in Makkah, Saudi Arabia. The age range of our study sample was set to 6-10 years, based on the mean age at which FPM eruption occurs and to cover the first phase of mixed dentition. The mean age of the study sample was 8.53 years, and the SD was 1.62 years, indicating a moderate spread across ages. When age was analyzed in terms of sex, the mean age of the male participants was slightly higher than that of the female participants, and the SDs reflected a similar distribution of ages within each sex category. In this study, the prevalence of patients diagnosed with EE of FPMs was 1.79%. This prevalence aligns with those reported in other studies conducted in Saudi Arabia and internationally. Among studies conducted in Saudi Arabia, our recorded prevalence was higher than that reported for impacted teeth (0.7%) in an Al-Madinah-based study [11]; however, it was lower than the prevalences observed by Aldowsari MK et al. in a study conducted in Riyadh (2.2%) [12] and by Yaseen SM in an Abha-based study (2.3%) [13]. At the international level, a study conducted in the United Arab Emirates (UAE) found that the prevalence of EE of FPMs in the studied sample was 4.1% [14]. In a Kuwait-based study, Jassim A et al. found a prevalence of 5.5% [15], and Moyers RE reported that 3% of American children presented with EE of FPMs in their study sample [16]. A retrospective study on the prevalence of EE of FPMs in Turkish children reported a prevalence of 2.65%, a figure that is in the same range as the prevalence found in the present study [17]. However, considerably higher and lower prevalences of EE of FPMs have also been found in other international studies. For example, in 2021, Hali H et al. reported a prevalence of 10.2% in their test population [18]. Meanwhile, studies conducted with Spanish and Thai children reported prevalences of EE of FPMs of 8.7% and 0.75%, respectively [2, 5]. The discrepancy in prevalence between our findings and those of other studies may be due to various factors, including ethnic and genetic differences in populations [19], and variations in sample sizes. Notably, smaller samples can diminish the reliability and validity of results.

Regarding gender, our results showed that EE of FPMs occurred predominantly in male patients compared to female patients, at prevalences of 1.1% and 0.69%, respectively, and a male-to-female ratio of 1.7:1. Nevertheless, there was no statistically significant difference between the two genders, which indicated that EE of FPMs affects females and males equally [7]. Our findings coincide with those reported in the literature [20-22]. In particular, our findings align with those of the local studies by Aldowsari MK et al. (conducted in Riyadh) and Yaseen SM (conducted in Abha) [12, 13]. In addition, at the international level, studies conducted in the UAE, Turkey, and Thailand also reported that EE of FPMs occurred more frequently in males than females, with no statistically significant difference between the two [2, 14, 17]. In our study, the degree of EE severity was determined during the examination of the panoramic radiographs and classified using a three-category system (mild, moderate, and severe). We used this three-category system rather than a four-category system to match the methodology used in other local studies and thus allow better comparison of results. This study revealed that the male patients had more severe EE than the female patients; however, the difference was not statistically significant (p = 0.063). It is worth noting that the p-value was close to the threshold, indicating a potential trend that may warrant further investigation. Our findings contradict those of Aldowsari MK et al., who reported a negative correlation between the severity of EE of FPMs and gender in their Riyadh-based study [12]. Our findings also oppose those of other studies that found female patients had more severe cases of EE compared to male patients [14, 23]. In support of the results of other studies described in the literature, no statistically significant difference in EE of FPMs was found in this study between the right and left sides of the dental arches [3, 6, 7, 17, 18]. Regarding jaw preference, the locations of the EE observed in our sample revealed a statistically significant difference and discrete pattern between the upper and lower arches. The prevalence in the maxillary arch was 1.59%, with 16 occurrences, while the prevalence in the mandibular arch was notably lower at 0.20%, with only two occurrences. Similar results were reported in most of the previous studies, which followed a similar sample size, study design, and methodological approach to this study [3, 6, 12]. Even though this study has produced valuable findings, since it is the first study on the prevalence and severity of EE of FPMs in children in Makkah, it has some limitations. These limitations include the study's cross-sectional nature, which limits the identification of any cause-and-effect relationship(s). Additionally, the sample was limited to children who sought dental treatment at two dental centers in Makkah and did not extend to the general community. Finally, the sample size was reduced due to the suboptimal quality of a large number of radiographs, which did not meet the inclusion criteria. This study and its outcomes are of interest to oral healthcare workers, including pediatric dentists, orthodontists, and general dentists. Clinicians should be aware of the prevalence of various clinical conditions, such as EE of FPMs, in their community, as early diagnosis is essential for providing optimum dental care and minimizing clinical complications that arise from delayed intervention.

## Conclusions

In this study sample, the prevalence of EE of FPMs among children in Makkah, Saudi Arabia, was found to be 1.79%, with male patients being more affected than female patients. Nevertheless, no statistically significant differences were observed in terms of gender. The maxillary arch was more affected than the mandibular arch, and no significant differences were found between the right and left sides.
